# Evolution of oral cancer treatment in an andalusian population sample: Rehabilitation with prosthetic obturation and removable partial prosthesis

**DOI:** 10.4317/jced.54023

**Published:** 2017-08-01

**Authors:** Rafael Flores-Ruiz, Lizette Castellanos-Cosano, María-Angeles Serrera-Figallo, Aida Gutiérrez-Corrales, Maribel Gonzalez-Martin, Jose-Luis Gutiérrez-Pérez, Daniel Torres-Lagares

**Affiliations:** 1 Department of Stomatology, School of Dentistry, University of Seville; 2 Oral and Maxillofacial Surgery Unit, Universitary Hospital Virgen del Rocío, Seville

## Abstract

**Background:**

Radical surgical resection as a treatment modality for oral cancer often leads to an extensive deficit in both the maxillary and mandibular levels, where the use of a palatal obturator prosthesis (POP) or removable partial denture (RPP). The aim of this study was to evaluate the treatment with POP and RPP in patients treated for oral cancer in the Unit of Prosthetic Rehabilitation of the University Hospital Virgen del Rocío in a period of 20 years.

**Material and Methods:**

Retrospective descriptive study during the years 1991 and 2011 analyzing oral cancer type, characteristics, treatment and follow-up. The sample consisted of patients whose tumor had previously been removed and who had been referred to the Oncological Rehabilitation Unit of the Oral and Maxillofacial Surgery Unit of the “Virgen del Rocío” University Hospital for rehabilitation. The inclusion criteria were patients whose underlying pathology was any type of neoplasia, which after its treatment had been referred to the aforementioned Oncological Prosthetic Rehabilitation unit.

**Results:**

Of the 45 patients included in our study, 15 patients were rehabilitated with palatal obturator (33.3%) and 5 patients with removable partial denture (11.1%). The mean age of the sample of patients with POP was 57.3 ± 9.23, while the mean age of the sample of patients with RPP was 58 ± 13.5. The most common underlying pathology in patients with POP was squamous cell carcinoma (60%), whereas in patients with RPP it was 100%. The most frequent location found among POP patients was the upper jaw, while in the PRP patients there was no predominant location. The univariate and multivariate logistic regressions did not show any statistically significant association between the independent variables age, sex, smoking habit and alcoholic habit with the dependent variable type of rehabilitating prosthesis.

**Conclusions:**

Based on our data, we can conclude that RPP is used in few cases of oncological rehabilitation. The POP has a greater use, as long as the defect in the bones of the facial middle third is limited.

** Key words:**Head and neck cancer, reconstructive surgery, Palatal obturators, removable partial dentures.

## Introduction

Radical surgical resection as an oral cancer treatment modality often results in an extensive maxillo-palatine deficit that can be repaired using reconstructive. Surgical techniques such as free microvascular flaps, pedicled flaps (1,2) or leaving a large area capable of healing in spontaneous form, aloud us to place a removable dental prosthesis (palatal obturators prostheis) or a permanent dental prosthesis (osteo-integrated implant) (3).

Reconstruction of the normal function of the maxillofacial region should include anatomical restoration of bone complex continuity, soft tissue reconstruction, preservation of mobility of the mandible, tongue, cheeks and soft palate, as well as labial competence, being these factors determinants in the success of the rehabilitation (4,5).

Although dental restorations through removable appliances have a low success rate in patients with extensive surgical treatment and who have received radiotherapy, it is the prefered choice when there is communication of anatomical cavities that are not naturally present (oral cavity and maxillary sinus, oral cavity and nasal fossa).

In the case of maxillary defects it is also frequent that a deviation of the buccal commissure lead to a collapse of the hemiarcate and the palpebral bag of the lip. The prosthetic device that is responsible for rehabilitating this type of maxillary defect is called obturator. There are different classifications that refer to maxillary defects in order to facilitate the choice of prosthesis design. The most commonly used classifications are Aramany (6,7), Spiro *et al.* (8) and Brown *et al.* (9). POP have two distinct parts; One, the obturator, which seals the vacuum provoked after surgery, and another, which restores the area of the palate, restores the volume of the altered alveolar ridge and the missing teeth to recover the occlusion (6,10).

The objective of this study was to evaluate the treatment with palatal obturator prosthesis and removable partial denture in patients treated for oral cancer at the Prosthetic Rehabilitation Unit of the Virgen del Rocío Universitary Hospital over a period of 20 years.

## Material and Methods

An observational retrospective descriptive study was carried out during the years 1991 and 2011 analyzing the type, characteristics, treatment and follow-up of oral cancer. The sample consisted of patients whose tumor had previously been removed and who had been referred for rehabilitation to the Oncology Rehabilitation Department of the Oral and Maxillofacial Surgery Unit of the “Virgen del Rocío” Universitary Hospital.

The inclusion criteria were patients whose underlying pathology was any type of neoplasia, which after treatment had been referred to the Oncology Rehabilitation department. Exclusion criteria were patients previously rehabilitated with implant prosthesis, rehabilitation treatment were contraindicated, and those that had not been able to obtain the information related to the variables studied.

The information obtained from the patients included in the study was entered into a data collection sheet. The variables studied were sex, age, tobacco, alcohol, baseline pathology, localization of squamous cell carcinoma, treatment performed and presence of relapse, of the total of the sample.

The present study was authorized by the Ethics Committee of the University of Seville, and the patients signed their consent for their clinical data to be used for scientific purposes, although the patients could not be identified. Statistical analysis of the variables studied was performed using the SPSS program. A chi-square test (for the study of the distribution of the different variables in the sample) and univariate and multivariate logistic regressions (to identify risk factors for oncological pathology) were carried out.

## Results

Of the initial analyzed sample of 60 patients, only 45 patients met the inclusion criteria. Of the sample analyzed, 31 subjects were men (68.9%) and 14 women (31.1%) (*p* = 0.0169). The mean age of the sample was 57 years ± 13.83.

Of the 45 patients included in our study, 15 patients were rehabilitated using palatal obturator prosthesis (33.3%) and 5 patients with removable partial denture (11.1%).

The pre-prosthetic variables studied in patients that were rehabilitated with a palatal obturator prosthesis (POP) were disaggrega-ted by age in  Table 1, while the post-surgical variables can be found in  Table 2.

**Table 1:**
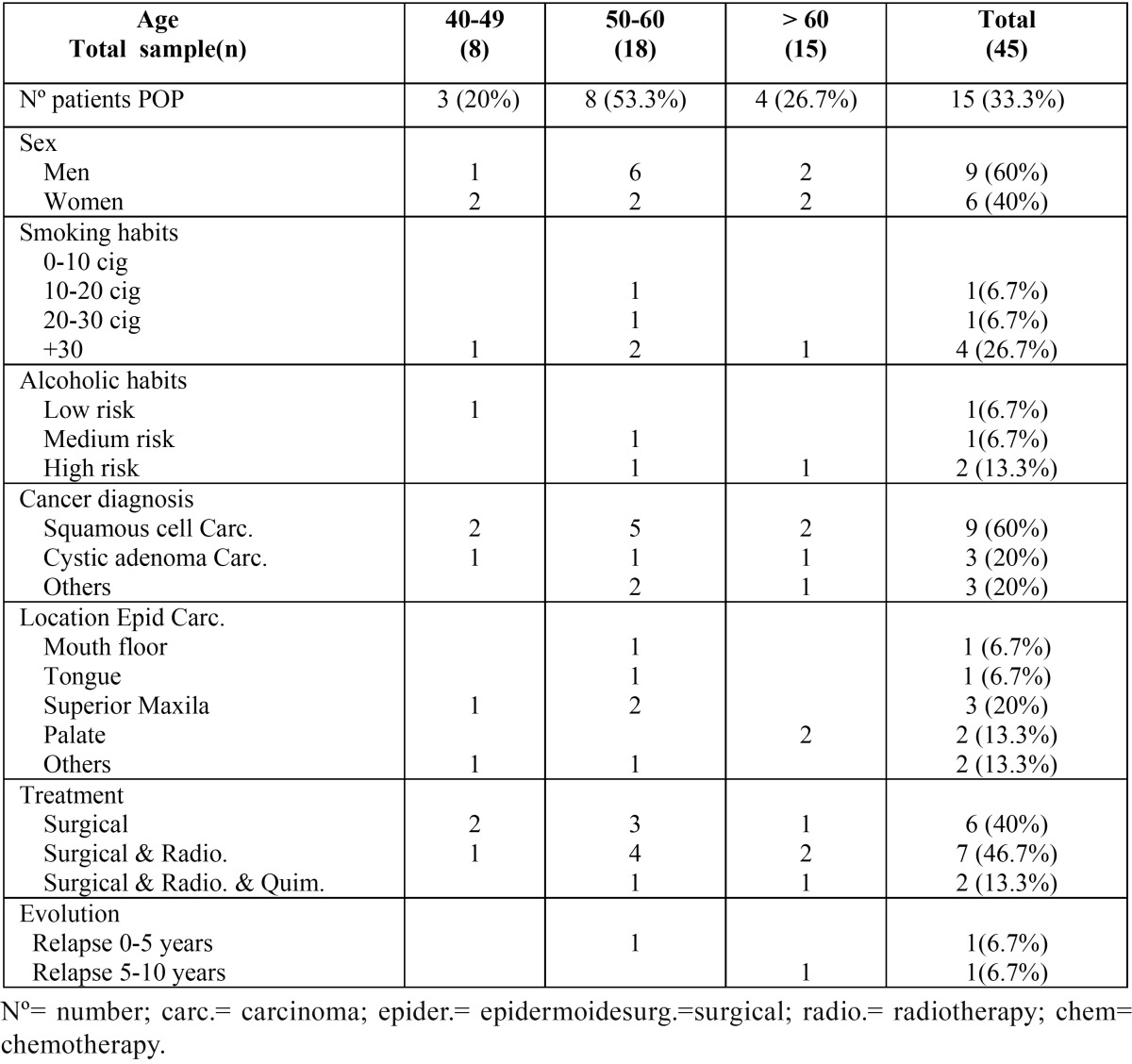
Pre-prosthetic variables studied by age in patients rehabilitated with palatal obturator prosthesis (POP).

**Table 2:**
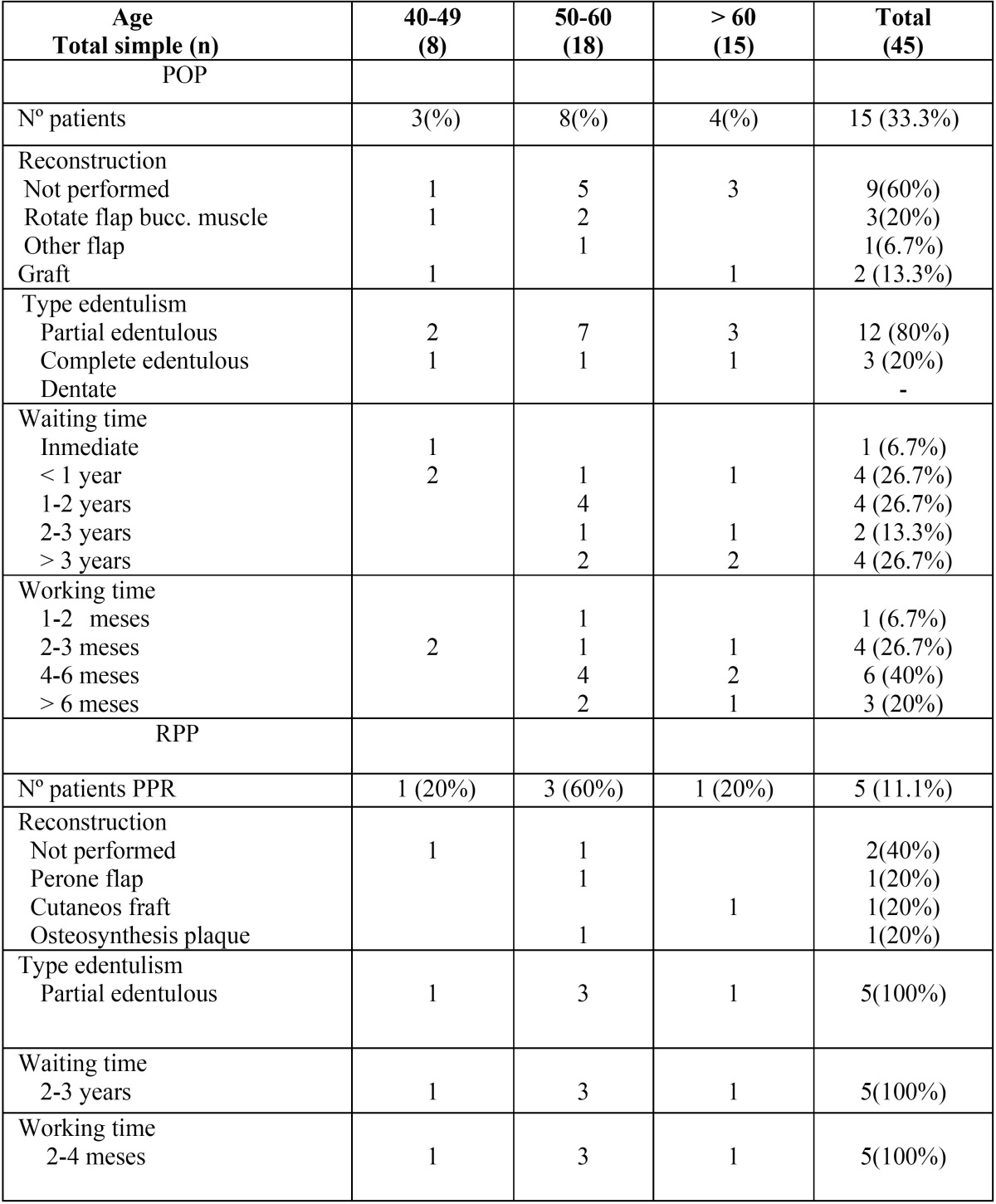
Post-surgical variables studied by age in patients rehabilitated with palatal obturator prostheses (POP) and removable partial prosthesis (RPP).

The pre-prosthetic variables studied in the patients that were rehabilitated with removable partial prostheses (RPP) are disaggregated by age in  Table 3, while the postsurgical variables can be found in  Table 2.

**Table 3:**
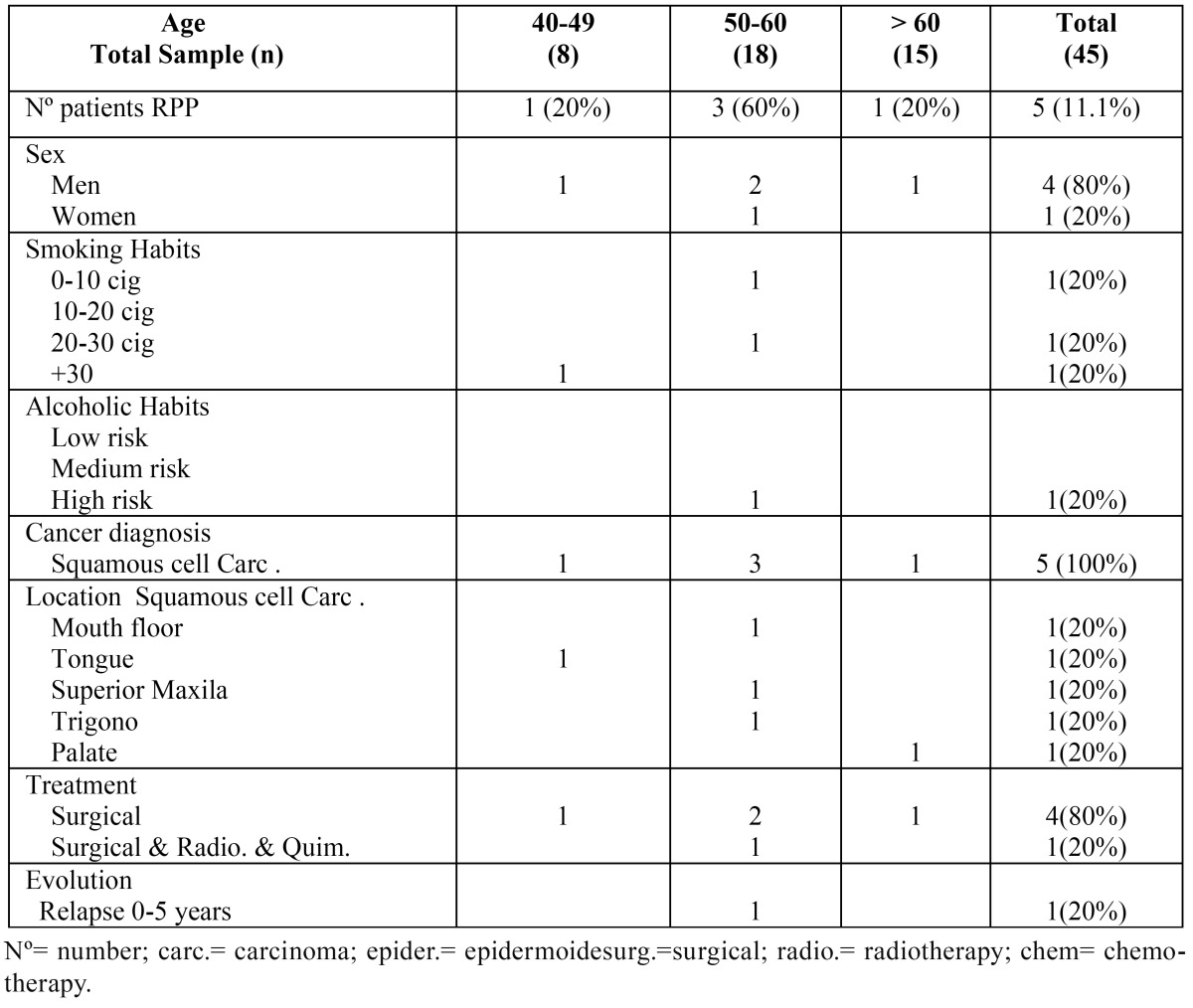
Pre-prosthetic variables studied by age in patients rehabilitated with removable partial prosthesis (RPP).

-Age

In the age range of 20 to 40 years, no patient was found to be rehabilitated with POP or RPP.

The mean age of the sample of patients with POP was 57.3 years ± 9.23, while the mean age of the sample of patients with RPP was 58 years ± 13.5. The breakdown of pre-prosthetic variables studied in patients with POPs are shown in  Table 1, while the pre-prosthetic variables studied in patients with PRP are found in  Table 3.

-Sex

Of the sample analyzed in patients with POP, 9 subjects were men (60%) and 6 women (40%) ( Table 1). In relation to patients with RPP, 4 were men (80%) and only one woman (20%) (Table 3).

-Smoking habit

Of the studied sample of patients with POP, 6 patients had a history of smoking (40%), of which 4 patients smoked more than 30 cigarettes per day (26.7%).

In patients rehabilitated with RPP, 60% had a history of smoking, of which only one patient smoked more than 30 cigarettes per day (20%).

-Alcoholic habit

Of the sample of patients with POP, 4 patients had a history of alcoholic habits (26.7%), of which 2 were high-risk drinkers (13.3%).

In patients rehabilitated with RPP, only 1 patient had a history of alcoholic habit, classified as a high-risk drinker (20%).

-Pathological diagnosis.

The most common underlying pathology in patients with POP was squamous cell carcinoma (60%) ( Table 1), whereas in patients with RPP it was 100% ( Table 3). The most frequent location found among patients with POP was the maxilla ( Table 1), whereas in the patients with RPP there was no predominant location ( Table 3).

-Type of treatment

The most frequent treatment modality in patients with POP was surgery with postoperative radiotherapy (46.7%), followed by surgery as the only treatment (40%) and only 13.3% of the cases were performed after surgery a combination of radiotherapy with chemotherapy ( Table 1). In patients with RPP, 80% received surgical treatment only and just 1 patient (20%) received a combination of radiotherapy and chemotherapy after surgery ( Table 3).

-Evolution

All patients had a minimum follow-up of 5 years, during this period only two patients in the sample of patients with POP had recurrence of the tumor pathology (13.3%), where one patient presented recurrence in the period from 0 to 5 years (6.7%) and another patient between 5-10 years (6.7%) ( Table 1). In the group of patients rehabilitated with RPP only one patient presented recurrence (0-5 years) ( Table 3).

-Surgical Reconstruction

Among the patients rehabilitated with POP, a reconstruction of the defect created by the surgical treatment was performed in 6 patients (40%). The most used was the rotated buccinator muscle flap with 20% (3 patients). No reconstruction was performed in 9 patients (60%) ( Table 2).

If we analyzed the patients rehabilitated with RPP, a reconstruction of the defect created by the surgical treatment was performed in 3 patients (60%). No reconstruction was performed in 2 patients (40%) ( Table 2).

-Type of edentulism

Of the 15 patients rehabilitated with POP, 80% were partial edentulous and 20% complete edentulous. In patients with RPP, 100% were partial edentulous ( Table 2).

-Wating time

Waiting times from the last phase of the oncological, surgical, radiotherapeutic or chemotherapeutic treatment to the preparation of the prosthesis were grouped into four blocks for information management.

Among patients rehabilitated with POP, 4 patients had a waiting period of less than one year (26.7%), 4 patients had a waiting period of one to two years (26.7%); 2 patients had a waiting period between two and three years (13.3%); And 4 patients had a waiting time of more than three years (26.7%) ( Table 2).

Among patients rehabilitated with RPP, all patients had an average waiting time of 2-3 years.

-Working time

The working time for the manufacture of POP was 1 to 2 months in one patient (6.7%), 2-3 months in 4 patients (26.7%), 4 to 6 months in 6 patients (40%) and more than 6 months in 3 patients (20%) ( Table 2). The working time for the preparation of RPP was 2-4 months in all patients.

The univariate and multivariate logistic regressions did not show any statistically significant association between the independent variables age, sex, smoking habit and alcoholic habit with the dependent variable type of rehabilitating prosthesis ( Table 4).

**Table 4:**
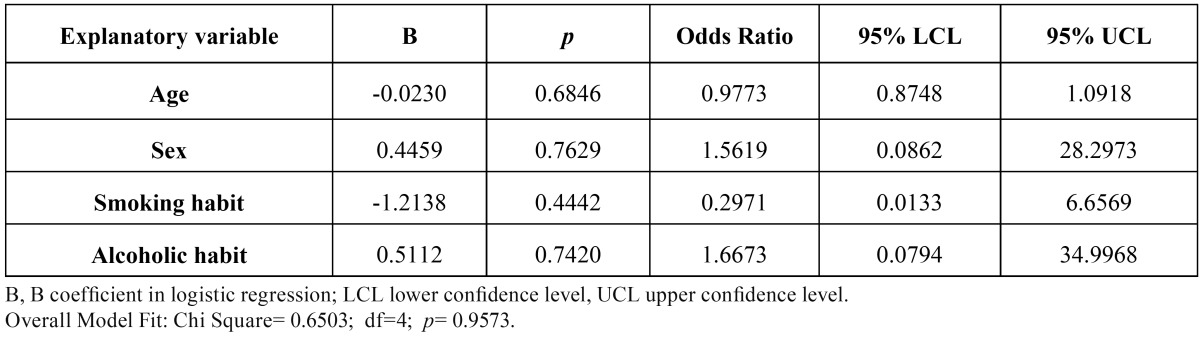
Multivariate logistic regressions of the association of independent variables, sex (0 = woman, 1 = male), age, smoking history (0 = no; 1 = yes) and alcoholic habits (0 = no; 1 = yes) on the dependent variable type of rehabilitating prosthesis (0 = PPR; 1 = POP).

## Discussion

The face is the part of the human body that expresses the character of the person, the feelings and the emotions. This is so, that patients who suffer an alteration in this region usually suffer a psychological alteration, with a feeling of inferiority and depression, because of diminished self-assessment. There is a moment in which the oncological pathology loses importance for the patient, and the imperative need for soft tissue reconstruction becomes important (11,12).

The morphology of the jaws has a functional and aesthetic role. Aesthetically, the maxillary bone is responsible for the projection of the nose, cheeks and the middle of the face. The palatino-maxillary defect can have serious consequences in terms of the relation between form and function: inability to chew and swallow disorders of phonation and important psychological implications (13).

The obturator prosthesis is a relevant prosthesis in the patient treated from oral cancer. Planning of this type of prosthesis should be carried out in the case of patients who present a communication after oncological surgery between two anatomical structures (previously not communicated), when other reconstructive alternatives are of extreme difficulty, or the result of them is expected to be unsatisfactory or unpredictable.

The obturator prosthesis offers several advantages: a) the possibility of immediately restoring the dentition without the need for additional surgery and b) allows optimal control of the oral cavity to identify possible recurrences of the disease. In the Rehabilitation Unit we observed that of 15 patients who were rehabilitated with obturator prosthesis, 60% of them preferred not undergo reconstruction surgery.

The moment of preparation of this prosthesis may be after oncologic surgery, if the dysfunction caused on the patient requires it, although for the preparation of the definitive prosthesis is advisable to wait for the maturation of the tissues. Tirelli *et al.* (3) in 3 isolated cases of patients with oral cancer, performed the rehabilitation treatment with definitive obturator prosthesis after 12 months, when the clinical recovery was complete and there were no recurrences of the disease, which is also recommended by other authors in the literature (fifteen). In our Unit, 26.7% of the patients were rehabilitated in less than a year, but when they were totally recovered from the disease.

Ortegon *et al.* (14) mentions various methods for retaining a complete bilateral obturator, which can be supported by using the remaining structures of the mouth, such as the posterior third of the soft palate, supporting the obturator with a lateral healing band, extra prosthesis orally into the nasal fossa or fix it with osseointegrated implants.

On the other hand, a vascularized graft can provide a permanent closure of the oral-nasal route. The reconstructive surgeon is face to diverse challenges such as recurrent disease in head and neck cancer patient, or undergoing multiple surgeries that leave the neck devoid of traditional recipient vessels. In addition, these patients have often received radiation therapy to the recipient vascular bed. The overall impact of radiation therapy on recipient vessels remains conflicting with some studies reporting negligible effects (15). Whereas others have shown this to be predictive of flap failure (16).

In such vessel depleted necks, many authors have described the use of interpositional vein grafts in order to close the distance between the free-flap and the nearest suitable recipient vessel. Unfortunately, vein grafts have been associated with an increased risk of thrombosis leading to flap complications and potential failure (17).

Authors such as Cordeiro *et al.* (6) observed systemic complications in 11.7% of patients and in 9.1% of patients undergoing a second exploration, which is necessary because the vessels of the free flap were compromised with partial necrosis in 1.8% (1).

The optimal technique for reconstruction of defects after surgical resection is still controversial. At the present time, there is no consensus regarding the optimal surgical management of maxillectomy defects. The surgeon must consider each defect and the needs of the individual patient when choosing the best suited reconstructive technique. Myriad of reconstructive options have been described in the literature including split thickness skin grafts, local flaps (palatal, buccal fat pad), regional flaps (deltopectoral, temporalis, submental), free-bone grafts and free-tissue transfer (osteocutaneous, fasciocutaneous , Myocutaneous, ‘sandwich’ wraps) (18).

Rogers et al. (19) performed studies to compare the repair through POP or free flap in relation to the function and quality of the recovery. Each technique has its advantages and disadvantages, however, the obturator prosthesis allows a satisfactory reconstruction and success is correlated in part to the extent of the extent of resection of the vertical and horizontal components. POP would be indicated when the resection is a quarter or less of the hard palate and a third or less of the soft palate. Also, found that POP patients were satisfied, although they were concerned about their appearance, having more pain and soreness in their mouths, being more aware of their upper teeth, more self-conscious and less satisfied with their upper dentures, and less satisfied with function. On the other hand, they did not observe statistically significant differences between the groups treated with free flaps or palatal obturators with respect to language, swallowing, appetite and psychological aspects.

As for the removable partial denture is a prosthesis indicated before the impossibility of performing another type of prosthesis, either because of lack of dental abutments or before no indication / possibility of placement of implants. For its preparation must be expected to complete healing of the tissues. In this study all patients had a waiting time of 2-3 years for the preparation of the same and an estimated work time between 2-4 months.

The changes undergone by the reconstruction of the oral cavity after resective surgery of the tumor, in addition to xerostomia produced by radiotherapy, make the retention of the removable prosthesis poor. This, together with soft tissue overload, which is usually very friable, makes the use of this type of prosthesis very restricted. Implants for patients who undergo oncologic surgery provide the possibility of retaining a prosthesis by themselves without the need for support in the patient’s weakened oral mucosa (20,21).

## Conclusions

RPP is used in few cases of oncological rehabilitation. The POP has a greater use, as long as the defect in the bones of the facial middle third is limited.
